# Antibiotic mixture effects on growth of the leaf-shredding stream detritivore *Gammarus fossarum*

**DOI:** 10.1007/s10646-017-1787-2

**Published:** 2017-03-11

**Authors:** Mirco Bundschuh, Torsten Hahn, Mark O. Gessner, Ralf Schulz

**Affiliations:** 1 Institute for Environmental Sciences, University of Koblenz-Landau, Landau Campus, Fortstrasse 7, 76829 Landau, Germany; 2 Department of Aquatic Sciences and Assessment, Swedish University of Agricultural Sciences, Box 7050, 75007 Uppsala, Sweden; 3 Fraunhofer Institute for Toxicology and Experimental Medicine, Nikolai-Fuchs-Strasse 1, 30625 Hannover, Germany; 4 Department of Experimental Limnology, Leibniz Institute of Freshwater Ecology and Inland Fisheries (IGB), Alte Fischerhütte 2, 16775 Stechlin, Germany; 5 Department of Ecology, Berlin Institute of Technology (TU Berlin), Ernst-Reuter-Platz 1, 10587 Berlin, Germany

**Keywords:** *Gammarus fossarum*, Food quality, Leaf-associated microbial community, Indirect effect, Physiological fitness

## Abstract

Pharmaceuticals contribute greatly to human and animal health. Given their specific biological targets, pharmaceuticals pose a significant environmental risk by affecting organisms and ecosystem processes, including leaf-litter decomposition. Although litter decomposition is a central process in forest streams, the consequences of exposure to pharmaceuticals remain poorly known. The present study assessed the impact of antibiotics as an important class of pharmaceuticals on the growth of the leaf-shredding amphipod *Gammarus fossarum* over 24 days. Exposure scenarios involved an antibiotic mixture (i.e. sulfamethoxazole, trimethoprim, erythromycin-H_2_O, roxithromycin, clarithromycin) at 0, 2 and 200 µg/L to assess impacts resulting from exposure to both water and food. The antibiotics had no effect on either leaf-associated fungal biomass or bacterial abundance. However, modification of leaf quality (e.g. through shifts in leaf-associated microbial communities) may have triggered faster growth of gammarids (assessed in terms of body mass gain) at the low antibiotic concentration relative to the control. At 200 µg/L, however, gammarid growth was not stimulated. This outcome might be due to a modified ability of the gut microflora to assimilate nutrients and carbon. Furthermore, the observed lack of increases in the diameter of the gammarids’ peduncles, despite an increase in gammarid mass, suggests antibiotic-induced effects in the moulting cycle. Although the processes responsible for the observed effects have not yet been identified, these results suggest a potential role of food-quality, gammarid gut microflora and alteration in the moulting cycle in mediating impacts of antibiotics on these detritivores and the leaf decomposition process in streams.

## Introduction

Pharmaceuticals contribute greatly to human and animal health and welfare. As a result, the global production of pharmaceuticals has rapidly grown over the last four decades (Bernhardt et al. 2017). However, these chemicals are often incompletely metabolized within the treated organisms and are only partly degraded during conventional wastewater treatment. This results in the release of significant amounts of pharmaceuticals and their metabolic products into surface waters, together with a broad range of other chemicals of industrial and domestic use (Hollender et al. 2009). After their release into a receiving water body, this mixture of chemicals (often referred to as micropollutants, Schwarzenbach et al. 2006) can affect local and downstream aquatic communities and the ecosystems processes to which the communities contribute (Englert et al. 2013). Since pharmaceuticals are specifically designed to exert high biological activity, for instance to treat bacterial infections, they are also likely to have adverse effects on microbes in aquatic ecosystems (Gessner and Tlili 2016).

Pharmaceuticals acting as antihistamines and antibiotics can affect primary production, microbial respiration and other biological processes (Jonsson et al. 2015; Rosi-Marshall et al. 2013; but see Wilson et al. 2004). Furthermore, the antibiotic ciprofloxacin affects the functional diversity (i.e., the ability to use different carbon sources) of leaf-associated microbial communities (Maul et al. 2006), with potentially negative implications for microbial leaf decomposition and thus nutrient cycling in streams. In addition, antibiotics can modify interactions between bacteria and fungi (i.e. aquatic hyphomycetes) colonising decomposing leaf material (Bundschuh et al. 2009). These impacts on leaf-associated microbial communities can propagate to primary consumers, including leaf-shredding invertebrates (shredder) such as gammarids (Zubrod et al. 2011).

Leaf-shredding gammarids show distinct feeding preference when given the choice between leaf material that has been microbially colonized (i.e. conditioned; sensu Cummins 1974) in either the absence or presence of antibiotics (Hahn and Schulz 2007). A leaf-shredding amphipod, *Gammarus fossarum* (Amphipoda, Crustacea), prefers leaf material conditioned during exposure to an antibiotic mixture over unexposed control leaves (Bundschuh et al. 2009). This preference was attributed to a higher fungal biomass and a putative shift in fungal community composition (Bundschuh et al. 2011) on leaf material conditioned in the presence of antibiotics. Fungi enhance the palatability of leaf litter for shredders (Bärlocher 1985; Graça et al. 2001; Rong et al. 1995) and can account for up to 100% to the growth of some leaf-shredding species feeding on conditioned leaves (Bärlocher and Sridhar 2014; Chung and Suberkropp 2009). Therefore, it is not surprising that fungicide-induced alterations in leaf-associated fungal biomass and community composition can affect the physiology (i.e. growth, lipid concentration and composition) of both *G. fossarum* (Zubrod et al. 2015a, b) and other shredders such as the isopod *Asellus aquaticus* (Feckler et al. 2016).

Despite the potential for contaminants to affect gammarid shredders, a genus frequently used in ecotoxicological investigations (Kunz et al. 2010), through both waterborne exposure and modifications of food quality, as reported for fungicides (Zubrod et al. 2015a), there is a lack of empirical investigations that elucidate these relationships. The present study assessed the impact of an antibiotic mixture composed of sulfamethoxazole, trimethoprim, erythromycin-H_2_O, roxithromycin and clarithromycin at sum concentrations of 2 and 200 µg/L via the direct (waterborne) and indirect (food-quality related) exposure pathways on the growth of the shredder *G. fossarum* in a 24-d bioassay. The selection of this antibiotic mixture at the two concentration levels was motivated by an earlier study (Bundschuh et al. 2009) indicating an impact at the higher concentration on the leaf-associated microbial community. Although the antibiotics used here are typically detected at levels an order of magnitude lower below wastewater treatment plant effluents than the individual concentrations applied in this study (e.g., Calamari et al. 2003), it is important to realize that natural mixtures of chemicals in receiving streams also comprise a multitude of additional (antibiotic) compounds (Kolpin et al. 2002).

## Materials and methods

### Preparation of leaf material

**Fig. 1:**
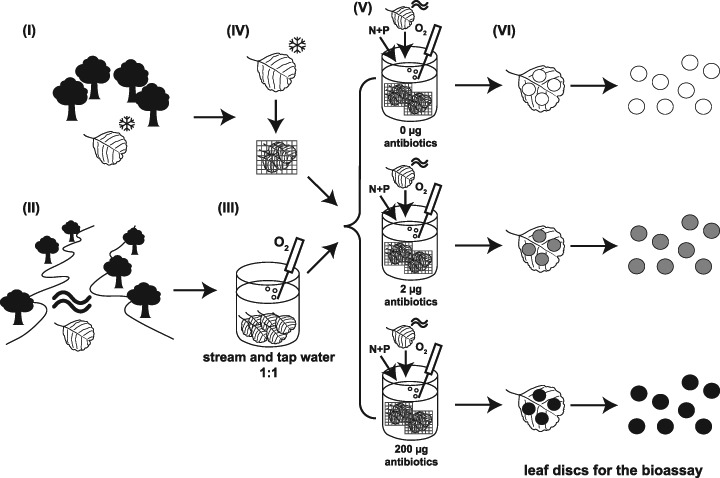
Schematic illustrating the preparation of leaf material used in gammarid growth experiments: (I) undecomposed black alder leaves were stored frozen (IV) until being placed in leaf bags. In parallel, (II) partly decomposed black alder leaves were collected from a local stream and (III) acclimated to laboratory conditions to serve as microbial inoculum. (V) This inoculum was transferred to aquaria filled with five litres of a stream and tap water mixture to promote microbial colonization of undecomposed leaf material in the presence of the antibiotic mixture at 0, 2 or 200 µg/L (indicated by *white*, *grey* and *black* discs, respectively). Finally, (VI) leaf discs were cut from the alder leaves after a conditioning period of 18 days and then offered as food to gammarids

Senescent but undecomposed black alder leaves (*Alnus glutinosa* (L.) Gaertn.) were collected shortly before leaf fall in October 2005 to serve as food for the test species. Leaves were picked from a group of trees near Mannheim, Germany (49° 32’ N, 8° 27’ E), and stored frozen at −20 °C until used (step I in Fig. 1). Freezing ensured that leaf material from a single batch and quality was available during the entire experiment. A microbial inoculum for the experiment was obtained by collecting partly decomposed black alder leaves from a small local stream (Rodenbach) near Mannheim, Germany (49° 33’ N, 8° 02’ E), in March 2006 (step II in Fig. 1). In the laboratory, the leaves were aerated for 14 days at 15 ± 1 °C in a 1:1 mixture of stream and tap water (see Table S1 for water quality) to acclimate the microbial community to the laboratory test conditions (Bundschuh et al. 2009; step III in Fig. 1). Subsequently, these leaves (25 g fresh mass) were transferred to circular 30-L aquaria filled with five litres of the stream and tap water mixture to promote microbial conditioning of the unconditioned leaf material. The 18-d conditioning period was repeated with new leaves every time food was renewed during the growth trials with gammarids.

The same aquaria were used for this conditioning step as for the gammarid growth trials (see below). Placing the unconditioned leaves in mesh bags (1.0 mm mesh size) allowed us to identify the individual conditioning runs and separate the newly conditioned leaves from those serving as inoculum (step IV in Fig. 1). After 18 days of conditioning (step V in Fig. 1) and 3 days before being fed to the gammarids, leaves were retrieved from the aquarium to cut leaf discs (2.0 cm diameter) with a cork borer (step VI in Fig. 1). These discs were dried at 60 °C to constant mass (about 24 h) and weighed to the nearest 0.01 mg. Subsequently, the leaf discs were soaked for 24 h in the stream and tap water mixture to prevent the leaves from floating on the water surface during the experiments.

### Handling of the test species *G. fossarum*

Specimens of *G. fossarum* were collected in April 2006 from the Rodenbach (see Bundschuh et al. 2009). In the laboratory, the collected animals were assigned to three size classes by using a passive underwater separation technique (Franke 1977). Males and females passing a 2.0-mm mesh screen but retained on a 1.7-mm mesh screen were used, which might have increase variability in the response variable but also increases relevance for effects at the population level. The cephalothorax length of these gammarids was between 1.2 and 1.6 mm. The animals were acclimatized for 1 week at 15 ± 1 °C in a 1:1 mixture of stream and tap water (see Table S1 for water quality) and total darkness. To initiate the 24-day experiment, 10 gammarids per aquarium were randomly and individually placed in cages (4 × 4 × 5 cm) made of 1.0-mm mesh screen. Two leaf discs were added. They originated from leaves conditioned in the same aquaria where the assays with gammarids were performed. Thus, gammarids were simultaneously subjected to antibiotics by exposure to both contaminated water and food. The water level in the cages was at least three centimetres during the experiment. Magnetic stirrers set at 200 rounds per minute ensured a water movement of 1 cm/s close to the aquarium wall. Seven replicate aquaria were set up for the antibiotic treatments (i.e., 2 and 200 µg/L) and the control, resulting in a total of 21 aquaria. Four additional cages containing two leaf discs but no gammarids were placed in each of the aquaria to determine leaf mass loss by microbial and physical processes. Water was renewed and food replaced every 6 days. Any leaf material remaining at these times was removed, dried and weighed as described above to determine leaf consumption by the gammarids (see Maltby et al. 2002; Zubrod et al. 2010).

Digital images of the gammarids were taken at the start and end of the experiment to determine the diameter of the peduncle (first segment of the antenna). Changes in the dry and fresh mass of gammarids were determined according to Pöckl (1992). Each specimen was weighed both before the experiment (fresh mass only) and at the end (fresh and dry mass). The initial dry mass of 90 additional gammarids was also determined to relate gammarid fresh mass to dry mass. This and the increase in peduncle diameter served as measures of gammarid growth. Data from specimens that died during the experiment were omitted from all analyses.

### Microbial analyses

Three leaf discs per replicate aquarium were taken after every second conditioning run to quantify bacterial abundance and fungal biomass. This resulted in seven independent replicates per sampling date and antibiotic concentration. Bacterial abundance was determined according to Buesing (2005): Following ultrasonication to detach bacterial cells from the leaf material, aliquots of the suspension were filtered onto aluminium oxide filters (0.2 µm pore size, Anodisc, Whatman, Beckenham, UK), and bacteria were stained with SYBRGreen II (Molecular Probes, Eugene, OR, USA). Digital pictures were taken under an epifluorescence microscope and used to determine bacterial abundance with image analysis software (Axio Scope.A1, AxioCam MRm, and AxioVision, Carl Zeiss MicroImaging). The bacterial counts were normalized to leaf dry mass determined for sets of leaf discs originating from the same leaf material.

Fungal biomass was estimated as ergosterol following the procedure described in Gessner (2005). Briefly, ergosterol was extracted in alkaline methanol (30 min, 80 °C). After purification of the extract by solid-phase extraction (SPE), ergosterol was quantified by measuring the absorbance at 282 nm using high-performance liquid chromatography (Jasco, Omnilab AG, Mettmenstetten, Switzerland) equipped with a LiChrospher-100-RP18 column (250 × 4 mm, 5-µm particle size; Merck, Dietikon, Switzerland). Ergosterol was converted to fungal biomass by assuming an average ergosterol concentration of 5.5 mg/g fungal dry mass (Gessner and Chauvet 1993).

### Chemicals and quantification of antibiotic

Clarithromycin (macrolide, Chemical Abstracts Services [CAS] 81103-11-9, purity >95%) was purchased from Molekula (Nienburg/Weser, Germany). Roxithromycin (macrolide, CAS 80214-83-1, purity >90%), sulfamethoxazole (sulfonamide, CAS 723-46-6, purity >98%), trimethoprim (CAS 738-70-5, purity >98.5%), and erythromycin (macrolide, CAS 114-07-8, purity >95%) were obtained from Sigma-Aldrich (Seelze, Germany). Erythromycin was transformed to erythromycin-H_2_O as described in McArdell et al. (2003). All other chemicals were purchased from Sigma-Aldrich (Seelze, Germany) or Roth (Karlsruhe, Germany). An antibiotic treatment at a high total concentration of 200 µg/L consisted of 40 µg/L of each of the five antibiotics in the mixture.

Four water samples were randomly collected from each of the treatments and analyzed by liquid chromatography tandem mass spectrometry (LC-MS/MS) to assess whether the nominal concentrations were achieved. Briefly, 50-mL subsamples were taken from each experimental unit both initially and immediately before water was exchanged (i.e., after 6 days). The water samples were stored at −20 °C until they were analysed without further sample concentration. The LC system used for the analyses was an HP 1100 (Agilent Technologies, Böblingen, Germany) equipped with a degasser (G1322A), pump (Agilent 1100 G1311A), autoinjector (Agilent 1100 G1329A), and column oven (Agilent 1100 G1316A). This system was connected to a liquid chromatography tandem mass spectrometry 4000 QTrap (Applied Biosystems, Foster City, CA, USA) detector. The HPLC column was a Chromolith performance RP-18e (100 × 4.6 mm, 5-µm particle size; Merck, Darmstadt, Germany). Antibiotics were separated along a nonlinear gradient starting with 100% solvent A and 0% solvent B and ending after 40 min with 0% A and 100% B. The flow rate was 400 µl/min. Solvent A was H_2_O:acetonitrile 90:10 (v/v) and solvent B was solvent A:acetonitrile 20:80 (v/v). The injection volume was 100 µl and the column temperature was 20 °C. Electrospray ionization with positive ionization was used for the mass-spectrometric detection of all antibiotics (Bundschuh et al. 2009). The measured concentrations of the antibiotics and the limits of quantification are summarized in Table 1.

**Table 1:** Limit of quantification (LOQ) and concentrations (mean ± standard error, *n* = 4) of the five antibiotics contained in the 200-µg/L antibiotic mixture (nominal concentration of each antibiotic: 40.0 µg/L) measured initially and 6 days later

Antibiotic	LOQ (µg/L)	Initial concentration (µg/L)	Concentration after 6 d (µg/L)
Erythromycin-H_2_O	0.28	41.5 ± 2.8	34.3 ± 3.5
Roxithromycin	0.12	29.6 ± 5.5	19.2 ± 2.7
Clarithromycin	0.16	33.7 ± 4.6	7.8 ± 2.1
Trimethoprim	0.09	33.6 ± 1.2	7.9 ± 0.6
Sulfamethoxazole	0.19	37.6 ± 4.7	4.6 ± 0.8

### Data analysis

The leaf mass consumed per day and per mg dry mass of surviving *G. fossarum* (C) was individually calculated for each replicate and week (Maltby et al. 2002) and then averaged over the study duration:

1 $$C = \frac{{\left( {{L_a} \times \left( {1 - k} \right)} \right) - {L_b}}}{{g \cdot t}}$$

where *L*_*a*_ = dry mass of the leaf discs after conditioning but before providing them as food to gammarids, *L*_*b*_ = dry mass of the conditioned leaf discs after 6 days of consumption by *G. fossarum*, *g* = dry mass of the gammarid at the end of the experiment, *t* = feeding time in days and *k* = is a correction factor for microbial and physical leaf mass loss determined by the following formula:

2 $$k = \frac{{{\sum} {\frac{{\left( {{L_c} - {L_d}} \right)}}{{{L_c}}}} }}{n}$$

where *L*_*c*_ = dry mass of conditioned leaf discs placed in cages without gammarids, *L*_*d*_ = dry mass of the same leaf discs but after 6 days of exposure, and *n* = number of replicates used per week. Moreover, the changes in the peduncle diameter, the fresh and dry mass of each gammarids over the 24-day study period were calculated and served as an indicator of growth. Differences in leaf associated bacterial abundance and fungal biomass, leaf consumption rate, increase in peduncle diameter and changes in fresh and dry mass were assessed by means of ANOVA followed by Dunnett’s tests for multiple comparisons, with the data from all cages within each replicate aquarium averaged prior to the analysis. The significance level was set at *p* < 0.05 for all tests.

## Results

**Fig. 2:**
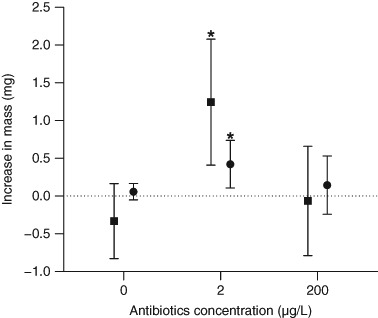
Mean (±95% CI) increases in the fresh (*squares*) and dry (*circles*) body mass of *G. fossarum* after 24 days of exposure to 0, 2 or 200 µg/L of an antibiotic mixture. The *dotted line* represents no difference in body mass. *Asterisks* indicate statistically significant deviations (*p* < 0.05) relative to the control based on Dunnett’s test for multiple comparisons

Bacterial abundances and fungal biomass associated with the leaf material fed to *G. fossarum* did not notably differ among treatments (Table 2). Similarly, the consumption of leaf material was largely unaffected by the antibiotic treatments (Tables 3, 4). Moreover, differences in the growth of gammarids measured as increase in fresh and dry mass was statistically significant across treatments as shown by the ANOVAs (Table 4). Higher growth was observed in water receiving 2 µg/L of the antibiotic mixture relative to the two other treatments (Fig. 2). A statistically significant effect was detected on the peduncle diameter (Table 4), showing an increase under control conditions yet no substantial change in the two antibiotic treatments, although a significant effect was noted at 2 µg/L (Table 3).

**Table 2:** Leaf-associated bacterial abundance and fungal biomass (mean ± standard deviation, *n* = 7) after 18 days of conditioning in 0, 2 and 200 µg/L of the five antibiotics

Concentration of the antibiotic mixture (µg/L)	Bacterial abundance (cells 10^11^/ mg leaf dry mass)	Fungal biomass (mg/g leaf dry mass)
0	4.2 ± 0.74	13.7 ± 8.5
2	3.5 ± 0.54	12.4 ± 5.1
200	3.9 ± 0.63	15.6 ± 8.3

**Table 3:** Leaf consumption (mg leaf dry mass/mg animal dry mass/day) by and increase in peduncle diameter (mm) of *G. fossarum* (mean ± standard deviation, *n* = 7) over the entire study duration of 24 days of exposure to 0, 2 and 200 µg/L of an antibiotics mixture

Concentration of antibiotic mixture (µg/L)	Leaf consumption (mg leaf dry mass/mg dry animal mass/day)	Increase in peduncle diameter (mm)
0	0.221 ± 0.020	0.019 ± 0.007
2	0.195 ± 0.038	0.001 ± 0.017*
200	0.229 ± 0.026	0.004 ± 0.014

The asterisk indicates a statistically significant deviation relative to the control based on Dunnett’s test for multiple comparisons

**Table 4:** Results of ANOVAs assessing differences among antibiotic treatments in leaf consumption by, and increases in the fresh mass, dry mass and peduncle diameter of *G. fossarum* over the entire study duration

Endpoint source of variation	df	SS	MS	F	P
Leaf consumption					
Antibiotic treatment	2	0.005	0.0023	2.69	0.095
Residuals	18	0.015	0.0008		
Fresh mass					
Antibiotic treatment	2	9.94	4.97	8.72	0.0023
Residuals	18	10.26	0.57		
Dry mass					
Antibiotic treatment	2	0.74	0.37	3.60	0.048
Residuals	18	1.84	0.10		
Peduncle diameter					
Antibiotic treatment	2	0.0014	0.0007	3.926	0.038
Residuals	18	0.0033	0.0002		

*df* degrees of freedom, *SS* sum of squares, *MS* mean squares

## Discussion

In contrast to Bundschuh et al. (2009), the present study did not detect large differences in either bacterial abundance or fungal biomass associated with leaf litter after 18 days of conditioning (Table 2). This discrepancy might be explained by different environmental conditions when the leaf material was conditioned in the presence or absence of antibiotics. In particular, the present study used a mixture of stream and tap water, whereas the earlier study relied on a well-defined test medium. This difference might have affected the leaf-associated microbial community during the conditioning process, because dissolved nitrogen availability was more than 10-fold lower than in the test medium used by Bundschuh et al. (2009) and may have influenced the colonisation dynamics of aquatic fungi (Fernandes et al. 2014), although the dissolved nitrogen levels were still relatively high. Moreover, the leaf material in the present study was more tightly packed (i.e. one freely floating leaf disc vs. several leaves packed in a 10 × 10 cm leaf bag) and the ratio of unconditioned leaf material to inoculum was higher during the conditioning process than in the earlier study, which could have influenced the colonisation of leaf material by bacteria and particularly fungi. This explanation accords with the roughly 50% lower fungal biomass in the present compared to the earlier study (Bundschuh et al. 2009). Although bacterial abundance and fungal biomass showed only small differences among the antibiotic treatments, differences in fungal community composition (which was not assessed in the present study) might have modified the nutritive value of the leaf material for leaf-shredding *G. fossarum* (e.g., Zubrod et al. 2015b).

Increased growth of *G. fossarum* in terms of fresh and dry mass was found at the low (i.e., 2 µg/L) but not the high concentration of the antibiotic mixture (Fig. 2; Table 4). At the same time, the leaf consumption of these organisms was not substantially affected relative to the control (~10% reduction relative to the control; Table 3). This suggests an increased palatability of the leaf material conditioned at the low antibiotic concentration (see also Bundschuh et al. 2009). In contrast, at the elevated antibiotic concentration of 200 µg/L, growth of the gammarids was not stimulated (Fig. 2). This pattern might indicate that a potential increase in leaf palatability was scattered by energetically costly detoxification mechanisms (Maltby 1999), which could not be compensated by increased food intake in conditions of high antibiotic concentrations (Table 3, Zubrod et al. 2015b).

Independent of this potential indirect (food-quality related) pathway, the gammarids were directly exposed to the antibiotic mixture dissolved in water during the entire 24-day test period. Antibiotics can alter the microflora in the gut of invertebrates and consequently their efficiency to assimilate nutrients and energy from the ingested food. For instance, Gorokhova et al. (2015) observed a lower diversity in the gut microflora that was associated with a lower assimilation efficiency in *Daphnia magna* exposed to one of the antibiotics assessed in the present study (trimethoprim) and at a similar concentration (250 µg/L). Such impacts on the gut microflora could explain the lack of growth observed for *G. fossarum* exposed to 200 µg/L of the antibiotic mixture, if the ability of *G. fossarum* to profit from a potentially higher food quality was lost at this high antibiotic concentration. In livestock production, antibiotics are often used as promoters that increase the rate and efficiency of growth (Cromwell 2002) at rather low doses. Similar mechanisms could have been effective at the low but not the high antibiotic concentration in the present study, for example by enhancing assimilation efficiency by reducing the importance of other, less beneficial or harmful community members of the microflora (as an example for chicken see Li et al. 2016).

Despite increased growth at the low antibiotic concentration, the peduncle diameter of gammarids did not change at either concentration, whereas it increased in the control organisms (Tables 3, 4). This deviation in the peduncle diameter might partly be due to effects on the moulting cycle of gammarids, suggesting that gammarids exposed to antibiotics failed to moult, despite the observed increase in body dry mass. If correct, this observation might indicate a moulting inhibition induced by the antibiotic mixture, an effect that has been previously reported for filarial nematodes exposed to the antibiotic tetracycline (Smith and Rajan 2000).

In conclusion, the present study design could not capture the effect pathways (food quality vs. waterborne exposure) and mechanisms responsible for the impacts observed at the physiological level (i.e., growth) of the leaf-shredding model species *G. fossarum*. Nonetheless, our finding that *G. fossarum* gained mass (Fig. 2) at a total antibiotic concentration close to concentrations measured in the field (2 µg/L; Kolpin et al. 2002), together with a possible impact on moulting (Table 3), deserves further attention. Since gammarids, like all arthropods, can only grow by moulting, increases in body mass but a failure to moult would have fatal consequences. These results call for a more systematic consideration of the implications of environmental release of antibiotics, as well as other pharmaceuticals such as antihistamines (Jonsson et al. 2015) or antidiabetics (Rosi-Marshall et al. 2013). Besides the indirect effect pathway mediated by food quality (as an example for fungicides see Zubrod et al. 2015c), impacts on the gut microflora and moulting success of invertebrates could be relevant and yet poorly understood effects of antibiotics in natural environments.

## Electronic supplementary material

Supplementary Information
