# Supplementary material for: Antibiotic mixture effects on growth of the leaf-shredding stream detritivore Gammarus fossarum
Source: Ecotoxicology. 2017 Mar 11;26(4):547–54. doi: 10.1007/s10646-017-1787-2 (PMC5420384; doi:10.1007/s10646-017-1787-2)
Supplement: Supplementary file 1 — Supplementary Information [file 10646_2017_1787_MOESM1_ESM.docx]

**Supporting Information**

**Do antibiotics modify the energetic foundation of heterotrophic food webs? A case study with *Gammarus fossarum***

Mirco Bundschuh^1,2^, Torsten Hahn^3^, Mark O. Gessner^4,5^ and Ralf Schulz^1^

^1^Institute for Environmental Sciences, University of Koblenz-Landau, Landau Campus, Fortstrasse 7, 76829 Landau, Germany

^2^Department of Aquatic Sciences and Assessment, Swedish University of Agricultural Sciences, Uppsala, Sweden

^3^Fraunhofer Institute for Toxicology and Experimental Medicine, Nikolai-Fuchs-Strasse 1, D-30625 Hannover, Germany

^4^Department of Experimental Limnology, Leibniz Institute of Freshwater Ecology and Inland Fisheries (IGB), Alte Fischerhütte 2, 16775, Stechlin, Germany

^5^Department of Ecology, Berlin Institute of Technology (TU Berlin), Ernst-Reuter-Platz 1, 10587, Berlin, Germany

*CORRESPONDING AUTHOR:

Mirco Bundschuh

Department of Aquatic Sciences and Assessment

Swedish University of Agricultural Sciences

P.O. Box 7050

750 07 Uppsala

Sweden

Phone: (+46) 72 516 97 68

Email: mirco.bundschuh@slu.se

Table S1: Mean values (n=3) of water quality variables immediately after and just before the water exchange. Nutrient concentrations and hardness were measured with Machery-Nagel test kits and oxygen saturation, conductivity and pH were measured with a WTW kit Multi 3401.

| Water quality  variable | Concentration immediately after water exchange | Concentration just before water exchange |
| --- | --- | --- |
| Nitrate (mg/L) | 8.3 | 12.2 |
| Nitrite (mg/L) | <0.02 | 0.02 |
| Ammonium (mg/L) | <0.02 | <0.02 |
| Phosphate (mg/L) | 0.30 | 0.44 |
| Conductivity (µS/cm) | 392 | 422 |
| pH | 8.07 | 8.42 |
| Hardness (°dH) | 12.0 | 12.4 |
| Oxygen saturation (%) | 80.3 | 90.2 |
